# Supplementary material for: Chronic shedding of a SARS-CoV-2 Alpha variant in wastewater
Source: BMC Genomics. 2024 Jan 13;25:59. doi: 10.1186/s12864-024-09977-7 (PMC10787452; doi:10.1186/s12864-024-09977-7)
Supplement: Supplementary file 1 — Additional file 1: Supplemental Table 1. MEGA and FastQC analyses of wastewater samples positive for an Alpha variant lineage. [file 12864_2024_9977_MOESM1_ESM.docx]

Supplemental Table 1. MEGA and FastQC analyses of wastewater samples positive for an Alpha variant lineage

| Sample | Spike reads^a^ | Read length^b^ | Poor quality sequences^c^ |
| --- | --- | --- | --- |
| VM 10-26-21 | 115,146 | 151 | 0 |
| VM 11-9-21 | 154,428 | 151 | 0 |
| VM 9-12-22 | 132,132 | 151 | 0 |
| VM 3-13-23 | 189,826 | 151 | 0 |
| VM 3-27-23 | 84,406 | 151 | 0 |
| VM 4-24-23 | 156,360 | 151 | 0 |
| VM 5-1-23 | 229,314 | 151 | 0 |
| VM 5-15-23 | 37,054 | 151 | 0 |
| CE 4-10-23 | 29,986 | 151 | 0 |
|  |  |  |  |

*a, Fastq files were aligned to reference surface glycoprotein (spike) and total spike reads were identified in MEGA. b,c FastQC was used to determine read length and the number of poor quality sequences.*
