# Supplementary material for: Chronic shedding of a SARS-CoV-2 Alpha variant in wastewater
Source: BMC Genomics. 2024 Jan 13;25:59. doi: 10.1186/s12864-024-09977-7 (PMC10787452; doi:10.1186/s12864-024-09977-7)
Supplement: Supplementary file 2 — Additional file 2: Supplemental Figure 1. Clustal Omega alignment of reconstructed 2021, 2022, and 2023 Spike proteins with reference and consensus Alpha variant Q.3 clinical sequence Spike proteins. [file 12864_2024_9977_MOESM2_ESM.docx]

Supplemental Figure 1

CLUSTAL O(1.2.4) multiple sequence alignment

Reference MFVFLVLLPLVSSQCVNLTTRTQLPPAYTNSFTRGVYYPDKVFRSSVLHSTQDLFLPFFS 60

VM_11-9-21 MFVFLVLLPLVSSQCVNLTTRTQLPPAYTNSFTRGVYYPDKVFRSSVLHSTQDLFLPFFS 60

Q.3 MFVFLVLLPLVSSQCVNLTTRTQLPPAYTNSFTRGVYYPDKVFRSSVLHSTQDLFLPFFS 60

VM-9-12-22 MFVFLVLLPLVFSQCVSLTTRTQLPPAYTNSFTRGVYYPDKVFRSSVLYSTQDLFLPFFS 60

VM_5-1-23 MFVFLVLLPLVSSQCVNLKTRTQLTPAYTNSFTRGVYYPDKVFRSSVLYSTQDLFLPFFS 60

*********** ****.*.***** ***********************:***********

Reference NVTWFHAIHVSGTNGTKRFDNPVLPFNDGVYFASTEKSNIIRGWIFGTTLDSKTQSLLIV 120

VM_11-9-21 NVTWFHA---SGTNGTKRFDNPVLPFNDGVYFASTEKSNIIRGWIFGTTLDSKTQSLLIV 117

Q.3 NVTWFHAI--SGTNGTKRFDNPVLPFNDGVYFASTEKSNIIRGWIFGTTLDSKTQSLLIV 118

VM-9-12-22 NVTRFQAI--SGTNGIKRFDNPVLPFNDGVYFASTEKSNIIRGWIFGTTLDSKTQSLLIV 118

VM_5-1-23 NVTRFQA-H-SGTNGIKRFDNPVLPFNDGVYFASTEKSNIIRGWIFGTTLDSKTQSLLIV 118

*** *:* ***** ********************************************

Reference NNATNVVIKVCEFQFCNDPFLGVYYHKNNKSWMESEFRVYSSANNCTFEYVSQPFLMDLE 180

VM_11-9-21 NNATNVVIKVCEFQFCNDPFLG--YHTNNKSWMESEFRVYSSANNCTFEYVSQPFLMDLE 175

Q.3 NNATNVVIKVCEFQFCNDPFLGV-YHKNNKSWMESEFRVYSSANNCTFEYVSQPFLMHLE 177

VM-9-12-22 NNATNVVIKVCEFQFCNDPFLGV-YHTNNKIWMESEFRVYSSANNCTFEYVSQPFLMDLE 177

VM_5-1-23 NNATNVVIKVCEFQFCNDPFLG--YHTNNKIWMESEFRVYSSANNCTFEYVSQPFLMDLE 176

********************** *.*** **************************.**

Reference GKQGNFKNLREFVFKNIDGYFKIYSKHTPINLVRDLPQGFSALEPLVDLPIGINITRFQT 240

VM_11-9-21 GKQGNFKNLREFVFKNIDGYFKIYSKHTPINLVRDLPQGFSALEPLVDLPIGINITRFQT 235

Q.3 GKQGNFKNLREFVFKNIDGYFKIYSKHTPINLVRDLPQGFSALEPLVDLPIGINITRFQT 237

VM-9-12-22 EKQGNFKNLREFVFKSIDGYFKIYSKHTPINLVRDLPQGFSALEPLVDLPIGINITRFQT 237

VM_5-1-23 EKQGNFKNLREFVFKSIDGYFKIYSKHTPINLVRDLPQGFSALEPLVDLPIGINITRFQT 236

**************.********************************************

Reference LLALHRSYLTPGDSSSGWTAGAAAYYVGYLQPRTFLLKYNENGTITDAVDCALDPLSETK 300

VM_11-9-21 LLALHRSYLTPGDSSSGWTAGAAAYYVGYLQPRTFLLKYNENGTITDAVDCALDPLSETK 295

Q.3 LLALHRSYLTPGDSSSGWTAGAAAYYVGYLQPRTFLLKYNENGTITDAVDCALDPLSETK 297

VM-9-12-22 LLALHRSYLTPGDSSSGWTAGAAAYYVGYLQPRTFLLKYNENGTITDAVDCALDPLSETK 297

VM_5-1-23 LLALHRSSLTPGDSSSDWTAGAAAYYVGYLQPRTFLLKYNENGTITDAVDCALDPLSETK 296

******* ********.*******************************************

Reference CTLKSFTVEKGIYQTSNFRVQPTESIVRFPNITNLCPFGEVFNATRFASVYAWNRKRISN 360

VM_11-9-21 CTLKSFTVEKGIYQTSNFRVQPTESIVRFPNITNLCPFGEVFNATRFASVYAWNRKRISN 355

Q.3 CTLKSFTVEKGIYQTSNFRVQPTESIVRFPNITNLCPFGEVFNATRFASVYAWNRKRISN 357

VM-9-12-22 CTLKSFTVEKGIYQTSNFRVQPTESIVRFPNITNLCPFGEVFNATRFASVYAWNRKRISN 357

VM_5-1-23 CTLKSFTVEKGIYQTSNFRVQPTESIVRFPNITNLCPFGEVFNATRFASVYAWNRKRISN 356

************************************************************

Reference CVADYSVLYNSASFSTFKCYGVSPTKLNDLCFTNVYADSFVIRGDEVRQIAPGQTGKIAD 420

VM_11-9-21 CVADYSVLYNSASFSTFKCYGVSPTKLNDLCFTNVYADSFVIRGDEVRQIAPGQTGKIAD 415

Q.3 CVADYSVLYNSASFSTFKCYGVSPTKLNDLCFTNVYADSFVIRGDEVRQIAPGQTGKIAD 417

VM-9-12-22 CVADYSVLYNSTSFSTFKCYGVSPTKLNDLCFTNVYADSFVIRGDEVRQIAPGQTGKIAD 417

VM_5-1-23 CVADYSVLYNSTSFSTFKCYGVSPTKLNDLCFTNVYADSFVIKGDEVRQIAPGQTGKIAD 416

***********:******************************:*****************

Reference YNYKLPDDFTGCVIAWNSNNLDSKVGGNYNYLYRLFRKSNLKPFERDISTEIYQAGSTPC 480

VM_11-9-21 YNYKLPDDFTGCVIAWNSNNLDSKVGGNHNYLYRLFRKSNLKPFERDISTEIYQAGSTPC 475

Q.3 YNYKLPDDFTGCVIAWNSNNLDSKVGGNYNYLYRLFRKSNLKPFERDISTEIYQAGSTPC 477

VM-9-12-22 YNYKLPDDFTGCVIAWNSNNLDSKVDGNNNYLFRLFRKSNLKPFERDISTEIYQAGSTPC 477

VM_5-1-23 YNYKLPDDFTGCVIAWNSNNLDS---GNNNYQFRLFRKSKLKPFERDISTEIYQAGNTPC 473

*********************** ** ** :******:****************.***

Reference NGVEGFNCYFPLQSYGFQPTNGVGYQPYRVVVLSFELLHAPATVCGPKKSTNLVKNKCVN 540

VM_11-9-21 NGVEGFNCYFPLQSYGFRPTYGVGYQPYRVVVLSFELLHAPATVCGPKKSTNLVKNKCVN 535

Q.3 NGVEGFNCYFPLQSYGFQPTYGVGYQPYRVVVLSFELLHAPATVCGPKKSTNLVKNKCVN 537

VM-9-12-22 NGVAGFNCYFPLQSYGFRPTYGVDHQPYRVVVLSFELLHAPATVCGPKKSTNLVKNKCVN 537

VM_5-1-23 NGVVGFNCYFPLKSYGFLPTYGVDHQPYRVVVLSFELLHAPATVCGPKKSTNLVKNKCVN 533

*** ********:**** ** **.:***********************************

Reference FNFNGLTGTGVLTESNKKFLPFQQFGRDIADTTDAVRDPQTLEILDITPCSFGGVSVITP 600

VM_11-9-21 FNFNGLTGTGVLTESNKKFLPFQQFGRDIDDTTDAVRDPQTLEILDITPCSFGGVSVITP 595

Q.3 FNFNGLTGTGVLTESNKKFLPFQQFGRDIDDTTDAVRDPQTLEILDITPCSFGGVSVITP 597

VM-9-12-22 FNFNGLTGTGVLTESNKKFLPFQQFGRDIDGTTDAVRDPQTLEILDVTPCSFGGVSVITP 597

VM_5-1-23 FNFNGLTGTGVLTESNKKFLPFQQFGRDIDGTTDAVRDPQTLEILDVTPCSFGGVSVITP 593

***************************** .***************:*************

Reference GTNTSNQVAVLYQDVNCTEVPVAIHADQLTPTWRVYSTGSNVFQTRAGCLIGAEHVNNSY 660

VM_11-9-21 GTNTSNQVAVLYQGVNCTEVPVAIHADQLTPTWRVYSTGSNVFQTRAGCLIGAEHVNNSY 655

Q.3 GTNTSNQVAVLYQGVNCTEVPVAIHADQLTPTWRVYSTGSNVFQTRAGCLIGAEHVNNSY 657

VM-9-12-22 GTNTSNQVAVLYQGVNCTEVPVAIHADQLTPTWRVYSTGSNVFQTRAGCLIGAEHVNNSY 657

VM_5-1-23 GTNTSNQVAVLYQGVNCTEVPVAIHADQLTPTWRVYSTGSNVFQTRAGCLIGAEHVNNSY 653

*************.**********************************************

Reference ECDIPIGAGICASYQTQTNSPRRARSVASQSIIAYTMSLGAENSVAYSNNSIAIPTNFTI 720

VM_11-9-21 ECDIPIGAGICASYQTQTNSHRRARSVASQSIIAYTMSLGAENSVAYSNNSIAIPINFTI 715

Q.3 ECDIPIGAGICASYQTQTNSHRRARSVASQSIIAYTMSLGAENSVAYSNNSIAIPINFTI 717

VM-9-12-22 ECDIPIGAGICASYQTQTNSHRRARSVASQSIISYTMSLGAENSVAYSNNSIAIPINFTI 717

VM_5-1-23 ECDIPIGAGICASYQTQTNSHRRARSVASQSIISYTMSLGAENSVAYSNNSIAIPTNFTI 713

******************** ************:********************* ****

Reference SVTTEILPVSMTKTSVDCTMYICGDSTECSNLLLQYGSFCTQLNRALTGIAVEQDKNTQE 780

VM_11-9-21 SVTTEILPVSMTKTSVDCTMYICGDSTECSNLLLQYGSFCTQLNRALTGIAVEQDKNTQE 775

Q.3 SVTTEILPVSMTKTSVDCTMYICGDSTECSNLLLQYGSFCTQLNRALTGIAVEQDKNTQE 777

VM-9-12-22 SVTTEILPVSMTKTSVDCTMYICGDSTECSNLLLQYGSFCTQLNRALTGIAAEQDKNTQE 777

VM_5-1-23 SVTTEILPVSMTKTSVDCTMYICGDSTECSNLLLQYGSFCTQLNRALTGIAAEQDKNTQE 773

***************************************************.********

Reference VFAQVKQIYKTPPIKDFGGFNFSQILPDPSKPSKRSFIEDLLFNKVTLADAGFIKQYGDC 840

VM_11-9-21 VFAQVKQIYKTPPIKDFGGFNFSQILPDPSKPSKRSFIEDLLFNKVTLADAGFIKQYGDC 835

Q.3 VFAQVKQIYKTPPIKDFGGFNFSQILPDPSKPSKRSFIEDLLFNKVTLADAGFIKQYGDC 837

VM-9-12-22 VFAQVKQIYKTPPIKDFGGFNFSQILPDPSKPSKRSFIEDLLFNKVTFADAGFIKQYGDC 837

VM_5-1-23 VFAQVKQIYKTPPIKDFGGFNFSQILPDPSKPSKRSFIEDLLFNKVTFADAGFIKQYGDC 833

***********************************************:************

Reference LGDIAARDLICAQKFNGLTVLPPLLTDEMIAQYTSALLAGTITSGWTFGAGAALQIPFAM 900

VM_11-9-21 LGDIAARDLICAQKFNGLTVLPPLLTDEMIAQYTSALLAGTITSGWTFGAGAALQIPFAM 895

Q.3 LGDIAARDLICAQKFNGLTVLPPLLTDEMIAQYTSALLAGTITSGWTFGAGAALQIPFAM 897

VM-9-12-22 LGDIAARDLICAQKFNGLTVLPPLLTDEMIAQYTSALLAGTITSGWTFGAGAALQIPFAM 897

VM_5-1-23 LGDIAARDLICAQKFNGLTVLPPLLTDEMIAQYTSALLAGTITSGWTFGAGAALQIPFAM 893

************************************************************

Reference QMAYRFNGIGVTQNVLYENQKLIANQFNSAIGKIQDSLSSTASALGKLQDVVNQNAQALN 960

VM_11-9-21 QMAYRFNGIGVTQNVLYENQKLIANQFNSAIGKIQDSLSSTASALGKLQDVVNQNAQALN 955

Q.3 QMAYRFNGIGVTQNVLYENQKLIANQFNSAIGKIQDSLSSTASALGKLQDVVNQNAQALN 957

VM-9-12-22 QMAYRFNGIGVTQNVLYENQKLIANQFNSAIGKIQDSLSSSASALGKLQDVVNQNAQALN 957

VM_5-1-23 QMAYRFNGIGVTQNVLYENQKLIANQFNSAIGKIQDSISSSASALGKLQDVVNQNAQALN 953

*************************************:**:*******************

Reference TLVKQLSSNFGAISSVLNDILSRLDKVEAEVQIDRLITGRLQSLQTYVTQQLIRAAEIRA 1020

VM_11-9-21 TLVKQLSSNFGAISSVLNDILARLDKVEAEVQIDRLITGRLQSLQTYVTQQLIRAAEIRA 1015

Q.3 TLVKQLSSNFGAISSVLNDILARLDKVEAEVQIDRLITGRLQSLQTYVTQQLIRAAEIRA 1017

VM-9-12-22 TLVKQLSSNFGAISSVLNDILARLDKVEAEVQIDRLITGRLQSLQTYVTQQLIRAAEIRA 1017

VM_5-1-23 TLVKQLSSNFGAISSVLNDILARLDKVEAEVQIDRLITGRLQSLQTYVTQQLIRAAEIRA 1013

*********************:**************************************

Reference SANLAATKMSECVLGQSKRVDFCGKGYHLMSFPQSAPHGVVFLHVTYVPAQEKNFTTAPA 1080

VM_11-9-21 SANLAATKMSECVLGQSKRVDFCGKGYHLMSFPQSAPHGVVFLHVTYVPAQEKNFTTAPA 1075

Q.3 SANLAATKMSECVLGQSKRVDFCGKGYHLMSFPQSAPHGVVFLHVTYVPAQEKNFTTAPA 1077

VM-9-12-22 SANLAATKMSECVLGQSKRVDFCGKGYHLMSFPQSAPHGVVFLHVTYVPAQEKNFTTAPA 1077

VM_5-1-23 SANLAATKMSECVLGQSKRVDFCGKGYHLMSFPQSAPHGVVFLHVTYVPAQEKNFTTAPA 1073

************************************************************

Reference ICHDGKAHFPREGVFVSNGTHWFVTQRNFYEPQIITTDNTFVSGNCDVVIGIVNNTVYDP 1140

VM_11-9-21 ICHDGKAHFPREGVFVSNGTHWFVTQRNFYEPQIITTDNTFVSGNCDVVIGIVNNTVYDP 1135

Q.3 ICHDGKAHFPREGVFVSNGTHWFVTQRNFYEPQIITTHNTFVSGNCDVVIGIVNNTVYDP 1137

VM-9-12-22 ICHDGKAHFPREGVFVSNGTHWFVTQRNFYEPQTITTHNTFVSGNCDVVIGIVNNTVYDP 1137

VM_5-1-23 ICHDGKAHFPREGVFVSNGTHWFVTQRNFYEPQIITTHNTFVSGNCDVVIGIVNNTVYDP 1133

********************************* ***.**********************

Reference LQPELDSFKEELDKYFKNHTSPDVDLGDISGINASVVNIQKEIDRLNEVAKNLNESLIDL 1200

VM_11-9-21 LQPELDSFKEELDKYFKNHTSPDVDLGDISGINASVVNIQKEIDRLNEVANNLNESLIDL 1195

Q.3 LQPELDSFKEELDKYFKNHTSPDVDLGDISGINASVVNIQKEIDRLNEVAKNLNESLIDL 1197

VM-9-12-22 LQPELDSFKEELDKYFKNHTSPNVDLGDIYGINASFVNIQKEIDRLNEVANNLNESLIDL 1197

VM_5-1-23 LQPELDSFKEELDKYFKNHTSPDVDLGDISGINASFVNIQKEIDRLNEVANNLNESLIDL 1193

**********************:****** *****.**************:*********

Reference QELGKYEQYIKWPWYIWLGFIAGLIAIVMVTIMLCCMTSCCSCLKGCCSCGSCCKFDEDD 1260

VM_11-9-21 QEFGKYEQYIKWPWYIWLGFIAGLIAIVMVTIMLCCMTSCCSCLKGCCSCGSCCKFDEDD 1255

Q.3 QELGKYEQYIKWPWYIWLGFIAGLIAIVMVTIMLCCMTSCCSCLKGCCSCGSCCKFDEDD 1257

VM-9-12-22 KELGKYEQYIKWPWYIWLGFIAGLIAIVMVTIMLCCMTSCCSCLKGCCSCGSCCKFDEDD 1257

VM_5-1-23 KELGKYEQYIKWPWYIWLGFIAGLIAIVMVTIMLCCMTSCCSCLKGCCSCGSCCKFDEDD 1253

:*:*********************************************************

Reference SEPVLKGVKLHYT 1273

VM_11-9-21 SEPVLKGVKLHYT 1268

Q.3 SEPVLKGVKLHYT 1270

VM-9-12-22 SEPVLKGVKLHYT 1270

VM_5-1-23 SEPVLKGVKLHYT 1266

*************

Supplemental Figure 1. Clustal Omega alignment of reconstructed 2021, 2022, and 2023 Spike proteins with reference and consensus Alpha variant Q.3 clinical sequence Spike proteins.
