# Supplementary material for: Chronic shedding of a SARS-CoV-2 Alpha variant in wastewater
Source: BMC Genomics. 2024 Jan 13;25:59. doi: 10.1186/s12864-024-09977-7 (PMC10787452; doi:10.1186/s12864-024-09977-7)
Supplement: Supplementary file 3 — Additional file 3: Supplemental Figure 2. Clustal Omega alignment of reconstructed 2021, 2022, and 2023 Spike proteins with reconstructed Spike protein from CE 4-10-23. Mutations unique to CE-4-10-23 are highlighted in grey. [file 12864_2024_9977_MOESM3_ESM.docx]

Supplemental Figure 2

CLUSTAL O(1.2.4) multiple sequence alignment

VM_11-9-21 MFVFLVLLPLVSSQCVNLTTRTQLPPAYTNSFTRGVYYPDKVFRSSVLHSTQDLFLPFFS 60

Reference MFVFLVLLPLVSSQCVNLTTRTQLPPAYTNSFTRGVYYPDKVFRSSVLHSTQDLFLPFFS 60

VM-9-12-22 MFVFLVLLPLVFSQCVSLTTRTQLPPAYTNSFTRGVYYPDKVFRSSVLYSTQDLFLPFFS 60

VM_5-1-23 MFVFLVLLPLVSSQCVNLKTRTQLTPAYTNSFTRGVYYPDKVFRSSVLYSTQDLFLPFFS 60

CE_2023 MFVFLVLLPLVSSQCVNLTTRTQSPPAYTNSFTRGVYYPDKVFRSSVLYSTQDLFLPFFS 60

*********** ****.*.**** ***********************:***********

VM_11-9-21 NVTWFHA---SGTNGTKRFDNPVLPFNDGVYFASTEKSNIIRGWIFGTTLDSKTQSLLIV 117

Reference NVTWFHAIHVSGTNGTKRFDNPVLPFNDGVYFASTEKSNIIRGWIFGTTLDSKTQSLLIV 120

VM-9-12-22 NVTRFQAI--SGTNGIKRFDNPVLPFNDGVYFASTEKSNIIRGWIFGTTLDSKTQSLLIV 118

VM_5-1-23 NVTRFQA-H-SGTNGIKRFDNPVLPFNDGVYFASTEKSNIIRGWIFGTTLDSKTQSLLIV 118

CE_2023 NVTRFQA---SGTNGIKRFDNPVLPFNDGVYFASTEKSNIIRGWIFGTTLDSKTQSLLIV 117

*** *:* ***** ********************************************

VM_11-9-21 NNATNVVIKVCEFQFCNDPFLG--YHTNNKSWMESEFRVYSSANNCTFEYVSQPFLMDLE 175

Reference NNATNVVIKVCEFQFCNDPFLGVYYHKNNKSWMESEFRVYSSANNCTFEYVSQPFLMDLE 180

VM-9-12-22 NNATNVVIKVCEFQFCNDPFLGV-YHTNNKIWMESEFRVYSSANNCTFEYVSQPFLMDLE 177

VM_5-1-23 NNATNVVIKVCEFQFCNDPFLG--YHTNNKIWMESEFRVYSSANNCTFEYVSQPFLMDLE 176

CE_2023 NNATNVVIKVCEFQFCNDPFLG--YHTNNKIWMESEFRVYSSANNCTFEYVSQPFLMDLE 175

********************** **.*** *****************************

VM_11-9-21 GKQGNFKNLREFVFKNIDGYFKIYSKHTPINLVRDLPQGFSALEPLVDLPIGINITRFQT 235

Reference GKQGNFKNLREFVFKNIDGYFKIYSKHTPINLVRDLPQGFSALEPLVDLPIGINITRFQT 240

VM-9-12-22 EKQGNFKNLREFVFKSIDGYFKIYSKHTPINLVRDLPQGFSALEPLVDLPIGINITRFQT 237

VM_5-1-23 EKQGNFKNLREFVFKSIDGYFKIYSKHTPINLVRDLPQGFSALEPLVDLPIGINITRFQT 236

CE_2023 EKQGNFKNLREFVFKSIDGYFKIYSKHTPINLVRDLPQGFSALEPLVDLPIGINITRFQT 235

**************.********************************************

VM_11-9-21 LLALHRSYLTPGDSSSGWTAGAAAYYVGYLQPRTFLLKYNENGTITDAVDCALDPLSETK 295

Reference LLALHRSYLTPGDSSSGWTAGAAAYYVGYLQPRTFLLKYNENGTITDAVDCALDPLSETK 300

VM-9-12-22 LLALHRSYLTPGDSSSGWTAGAAAYYVGYLQPRTFLLKYNENGTITDAVDCALDPLSETK 297

VM_5-1-23 LLALHRSSLTPGDSSSDWTAGAAAYYVGYLQPRTFLLKYNENGTITDAVDCALDPLSETK 296

CE_2023 LLALYRSYLTPGDSSSDWTAGAAAYYVGYLQPRTFLLKYNENGTITDAVDCALDPLSETK 295

****:** ********.*******************************************

VM_11-9-21 CTLKSFTVEKGIYQTSNFRVQPTESIVRFPNITNLCPFGEVFNATRFASVYAWNRKRISN 355

Reference CTLKSFTVEKGIYQTSNFRVQPTESIVRFPNITNLCPFGEVFNATRFASVYAWNRKRISN 360

VM-9-12-22 CTLKSFTVEKGIYQTSNFRVQPTESIVRFPNITNLCPFGEVFNATRFASVYAWNRKRISN 357

VM_5-1-23 CTLKSFTVEKGIYQTSNFRVQPTESIVRFPNITNLCPFGEVFNATRFASVYAWNRKRISN 356

CE_2023 CTLKSFTVEKGIYQTSNFRVQPTESIVRFPNITNLCPFGEVFNATRFASVYAWNRKRISN 355

************************************************************

VM_11-9-21 CVADYSVLYNSASFSTFKCYGVSPTKLNDLCFTNVYADSFVIRGDEVRQIAPGQTGKIAD 415

Reference CVADYSVLYNSASFSTFKCYGVSPTKLNDLCFTNVYADSFVIRGDEVRQIAPGQTGKIAD 420

VM-9-12-22 CVADYSVLYNSTSFSTFKCYGVSPTKLNDLCFTNVYADSFVIRGDEVRQIAPGQTGKIAD 417

VM_5-1-23 CVADYSVLYNSTSFSTFKCYGVSPTKLNDLCFTNVYADSFVIKGDEVRQIAPGQTGKIAD 416

CE_2023 CVADYSVLYNSTSFSTFKCYGVSPTKLNDLCFTNVYADSFVIRGDEVRQIAPGQTGKIAD 415

***********:******************************:*****************

VM_11-9-21 YNYKLPDDFTGCVIAWNSNNLDSKVGGNHNYLYRLFRKSNLKPFERDISTEIYQAGSTPC 475

Reference YNYKLPDDFTGCVIAWNSNNLDSKVGGNYNYLYRLFRKSNLKPFERDISTEIYQAGSTPC 480

VM-9-12-22 YNYKLPDDFTGCVIAWNSNNLDSKVDGNNNYLFRLFRKSNLKPFERDISTEIYQAGSTPC 477

VM_5-1-23 YNYKLPDDFTGCVIAWNSNNLDS---GNNNYQFRLFRKSKLKPFERDISTEIYQAGNTPC 473

CE_2023 YNYKLPDDFTGCVIAWNSNNLDS-A-GNNNYLFRLFRKSNLKPFERDISTEIYQAGSTPC 472

*********************** ** ** :******:****************.***

VM_11-9-21 NGVEGFNCYFPLQSYGFRPTYGVGYQPYRVVVLSFELLHAPATVCGPKKSTNLVKNKCVN 535

Reference NGVEGFNCYFPLQSYGFQPTNGVGYQPYRVVVLSFELLHAPATVCGPKKSTNLVKNKCVN 540

VM-9-12-22 NGVAGFNCYFPLQSYGFRPTYGVDHQPYRVVVLSFELLHAPATVCGPKKSTNLVKNKCVN 537

VM_5-1-23 NGVVGFNCYFPLKSYGFLPTYGVDHQPYRVVVLSFELLHAPATVCGPKKSTNLVKNKCVN 533

CE_2023 NGVVGFNCYFPLKSYGFLPTYGVDHQPYRVVVLSFELLHAPATVCGPKKSTNLVKNKCVN 532

*** ********:**** ** **.:***********************************

VM_11-9-21 FNFNGLTGTGVLTESNKKFLPFQQFGRDIDDTTDAVRDPQTLEILDITPCSFGGVSVITP 595

Reference FNFNGLTGTGVLTESNKKFLPFQQFGRDIADTTDAVRDPQTLEILDITPCSFGGVSVITP 600

VM-9-12-22 FNFNGLTGTGVLTESNKKFLPFQQFGRDIDGTTDAVRDPQTLEILDVTPCSFGGVSVITP 597

VM_5-1-23 FNFNGLTGTGVLTESNKKFLPFQQFGRDIDGTTDAVRDPQTLEILDVTPCSFGGVSVITP 593

CE_2023 FNFNGLTGTGVLTESNKKFLPFQQFGRDIDGTTDAVRDPQTLEILDVTPCSFGGVSVITP 592

***************************** .***************:*************

VM_11-9-21 GTNTSNQVAVLYQGVNCTEVPVAIHADQLTPTWRVYSTGSNVFQTRAGCLIGAEHVNNSY 655

Reference GTNTSNQVAVLYQDVNCTEVPVAIHADQLTPTWRVYSTGSNVFQTRAGCLIGAEHVNNSY 660

VM-9-12-22 GTNTSNQVAVLYQGVNCTEVPVAIHADQLTPTWRVYSTGSNVFQTRAGCLIGAEHVNNSY 657

VM_5-1-23 GTNTSNQVAVLYQGVNCTEVPVAIHADQLTPTWRVYSTGSNVFQTRAGCLIGAEHVNNSY 653

CE_2023 GTNTSNQVAVLYQGVNCTEVPVAIHADQLTPTWRVYSTGSNVFQTRAGCLIGAEHVNNSY 652

*************.**********************************************

VM_11-9-21 ECDIPIGAGICASYQTQTNSHRRARSVASQSIIAYTMSLGAENSVAYSNNSIAIPINFTI 715

Reference ECDIPIGAGICASYQTQTNSPRRARSVASQSIIAYTMSLGAENSVAYSNNSIAIPTNFTI 720

VM-9-12-22 ECDIPIGAGICASYQTQTNSHRRARSVASQSIISYTMSLGAENSVAYSNNSIAIPINFTI 717

VM_5-1-23 ECDIPIGAGICASYQTQTNSHRRARSVASQSIISYTMSLGAENSVAYSNNSIAIPTNFTI 713

CE_2023 ECDIPIGAGICASYQTQTNSHRRARSVASQSIIAYTMSLGAENSVAYSNNSIAIPTNFTI 712

******************** ************:********************* ****

VM_11-9-21 SVTTEILPVSMTKTSVDCTMYICGDSTECSNLLLQYGSFCTQLNRALTGIAVEQDKNTQE 775

Reference SVTTEILPVSMTKTSVDCTMYICGDSTECSNLLLQYGSFCTQLNRALTGIAVEQDKNTQE 780

VM-9-12-22 SVTTEILPVSMTKTSVDCTMYICGDSTECSNLLLQYGSFCTQLNRALTGIAAEQDKNTQE 777

VM_5-1-23 SVTTEILPVSMTKTSVDCTMYICGDSTECSNLLLQYGSFCTQLNRALTGIAAEQDKNTQE 773

CE_2023 SVTTEILPVSMTKTSVDCTMYICGDSTECSNLLLQYGSFCTQLNRALTGIAAEQDKNTQE 772

***************************************************.********

VM_11-9-21 VFAQVKQIYKTPPIKDFGGFNFSQILPDPSKPSKRSFIEDLLFNKVTLADAGFIKQYGDC 835

Reference VFAQVKQIYKTPPIKDFGGFNFSQILPDPSKPSKRSFIEDLLFNKVTLADAGFIKQYGDC 840

VM-9-12-22 VFAQVKQIYKTPPIKDFGGFNFSQILPDPSKPSKRSFIEDLLFNKVTFADAGFIKQYGDC 837

VM_5-1-23 VFAQVKQIYKTPPIKDFGGFNFSQILPDPSKPSKRSFIEDLLFNKVTFADAGFIKQYGDC 833

CE_2023 VFAQVKQIYKTPPIKDFGGFNFSQILPDPSKPSKRSFIEDLLFNKVTFADAGFIKQYGDC 832

***********************************************:************

VM_11-9-21 LGDIAARDLICAQKFNGLTVLPPLLTDEMIAQYTSALLAGTITSGWTFGAGAALQIPFAM 895

Reference LGDIAARDLICAQKFNGLTVLPPLLTDEMIAQYTSALLAGTITSGWTFGAGAALQIPFAM 900

VM-9-12-22 LGDIAARDLICAQKFNGLTVLPPLLTDEMIAQYTSALLAGTITSGWTFGAGAALQIPFAM 897

VM_5-1-23 LGDIAARDLICAQKFNGLTVLPPLLTDEMIAQYTSALLAGTITSGWTFGAGAALQIPFAM 893

CE_2023 LGDIAARDLICAQKFNGLTVLPPLLTDEMIAQYTSALLAGTITSGWTFGAGAALQIPFAM 892

************************************************************

VM_11-9-21 QMAYRFNGIGVTQNVLYENQKLIANQFNSAIGKIQDSLSSTASALGKLQDVVNQNAQALN 955

Reference QMAYRFNGIGVTQNVLYENQKLIANQFNSAIGKIQDSLSSTASALGKLQDVVNQNAQALN 960

VM-9-12-22 QMAYRFNGIGVTQNVLYENQKLIANQFNSAIGKIQDSLSSSASALGKLQDVVNQNAQALN 957

VM_5-1-23 QMAYRFNGIGVTQNVLYENQKLIANQFNSAIGKIQDSISSSASALGKLQDVVNQNAQALN 953

CE_2023 QMAYRFNGIGVTQNVLYENQKLIANQFNSAIGKIQDSLSSSASALGKLQDVVNQNAQALN 952

*************************************:**:*******************

VM_11-9-21 TLVKQLSSNFGAISSVLNDILARLDKVEAEVQIDRLITGRLQSLQTYVTQQLIRAAEIRA 1015

Reference TLVKQLSSNFGAISSVLNDILSRLDKVEAEVQIDRLITGRLQSLQTYVTQQLIRAAEIRA 1020

VM-9-12-22 TLVKQLSSNFGAISSVLNDILARLDKVEAEVQIDRLITGRLQSLQTYVTQQLIRAAEIRA 1017

VM_5-1-23 TLVKQLSSNFGAISSVLNDILARLDKVEAEVQIDRLITGRLQSLQTYVTQQLIRAAEIRA 1013

CE_2023 TLVKQLSSNFGAISSVLNDILARLDKVEAEVQIDRLITGRLQSLQTYVTQQLIRAAEIRA 1012

*********************:**************************************

VM_11-9-21 SANLAATKMSECVLGQSKRVDFCGKGYHLMSFPQSAPHGVVFLHVTYVPAQEKNFTTAPA 1075

Reference SANLAATKMSECVLGQSKRVDFCGKGYHLMSFPQSAPHGVVFLHVTYVPAQEKNFTTAPA 1080

VM-9-12-22 SANLAATKMSECVLGQSKRVDFCGKGYHLMSFPQSAPHGVVFLHVTYVPAQEKNFTTAPA 1077

VM_5-1-23 SANLAATKMSECVLGQSKRVDFCGKGYHLMSFPQSAPHGVVFLHVTYVPAQEKNFTTAPA 1073

CE_2023 SANLAATKMSECVLGQSKRVDFCGKGYHLMSFPQSAPHGVVFLHVTYVPAQEKNFTTAPA 1072

************************************************************

VM_11-9-21 ICHDGKAHFPREGVFVSNGTHWFVTQRNFYEPQIITTDNTFVSGNCDVVIGIVNNTVYDP 1135

Reference ICHDGKAHFPREGVFVSNGTHWFVTQRNFYEPQIITTDNTFVSGNCDVVIGIVNNTVYDP 1140

VM-9-12-22 ICHDGKAHFPREGVFVSNGTHWFVTQRNFYEPQTITTHNTFVSGNCDVVIGIVNNTVYDP 1137

VM_5-1-23 ICHDGKAHFPREGVFVSNGTHWFVTQRNFYEPQIITTHNTFVSGNCDVVIGIVNNTVYDP 1133

CE_2023 ICHDGKAHFPREGVFVSNGTHWFVTQRNFYEPQIITTHNTFVSGNCDVVIGIVNNTVYDP 1132

********************************* ***.**********************

VM_11-9-21 LQPELDSFKEELDKYFKNHTSPDVDLGDISGINASVVNIQKEIDRLNEVANNLNESLIDL 1195

Reference LQPELDSFKEELDKYFKNHTSPDVDLGDISGINASVVNIQKEIDRLNEVAKNLNESLIDL 1200

VM-9-12-22 LQPELDSFKEELDKYFKNHTSPNVDLGDIYGINASFVNIQKEIDRLNEVANNLNESLIDL 1197

VM_5-1-23 LQPELDSFKEELDKYFKNHTSPDVDLGDISGINASFVNIQKEIDRLNEVANNLNESLIDL 1193

CE_2023 LQPELDSFKEELDKFFKNHTSPDVDLGDISGINASFVNIQKEIDRLNEVANNLNESLIDL 1192

**************:*******:****** *****.**************:*********

VM_11-9-21 QEFGKYEQYIKWPWYIWLGFIAGLIAIVMVTIMLCCMTSCCSCLKGCCSCGSCCKFDEDD 1255

Reference QELGKYEQYIKWPWYIWLGFIAGLIAIVMVTIMLCCMTSCCSCLKGCCSCGSCCKFDEDD 1260

VM-9-12-22 KELGKYEQYIKWPWYIWLGFIAGLIAIVMVTIMLCCMTSCCSCLKGCCSCGSCCKFDEDD 1257

VM_5-1-23 KELGKYEQYIKWPWYIWLGFIAGLIAIVMVTIMLCCMTSCCSCLKGCCSCGSCCKFDEDD 1253

CE_2023 KELGKYEQYIKWPWYIWLGFIAGLIAIVMVTIMLCCMTSCCSCLKGCCSCGSCCKFDEDD 1252

:*:*********************************************************

VM_11-9-21 SEPVLKGVKLHYT 1268

Reference SEPVLKGVKLHYT 1273

VM-9-12-22 SEPVLKGVKLHYT 1270

VM_5-1-23 SEPVLKGVKLHYT 1266

CE_2023 SEPVLKGVKLHYT 1265

*************

Supplemental Figure 2. Clustal Omega alignment of reconstructed 2021, 2022, and 2023 Spike proteins with reconstructed Spike protein from CE 4-10-23. Mutations unique to CE-4-10-23 are highlighted in grey.
